# Family or caregiver outcomes after critical illness (FOCUS): a protocol for a core outcome set development study

**DOI:** 10.1136/bmjopen-2026-117825

**Published:** 2026-06-23

**Authors:** Claire Brown, Peter Hartley, Alexandra Malyon, Bronwen Connolly, Nazir I Lone, Joanne McPeake

**Affiliations:** 1 Cambridge University Hospitals NHS Foundation Trust, Cambridge, UK; 2 The Healthcare Improvement Studies Institute, University of Cambridge, Cambridge, UK; 3 Wellcome-Wolfson Institute for Experimental Medicine, Queen’s University Belfast, Belfast, UK; 4 Department of Physiotherapy, The University of Melbourne, Melbourne, Victoria, Australia; 5 The Usher Institute, University of Edinburgh, Edinburgh, UK

**Keywords:** Caregivers, Clinical Protocols, Delphi Technique, Adult intensive & critical care

## Abstract

**Introduction:**

Family members of survivors of critical illness are known to experience adverse outcomes in the months following the critical care admission, which can impact their physical, social and emotional health. This protocol describes the Family or Caregiver Outcomes after critical illness (FOCUS) study, which aims to develop a core outcome set (COS) for trials involving family members of critical care patients across the continuum of care.

**Methods and analysis:**

A modified Delphi consensus process with researcher, clinician and patient/caregiver stakeholder groups and consensus meetings for ratification of findings, resolving uncertainty and developing an action plan for COS implementation.

**Ethics and dissemination:**

The FOCUS COS will inform relevant stakeholders about important outcomes for family members of critical care patients and may enhance the future design and conduct of trials in this area. This study has been approved by the University of Cambridge Psychology Research Ethics Committee. The COS will be disseminated through peer-reviewed publications and engagement with key stakeholders.

**Trial registration number:**

COMET database (https://comet-initiative.org/Studies/Details/1977).

**STRENGTHS AND LIMITATIONS OF THIS STUDY**

- This core outcome set protocol will be developed according to the Core Outcome Measures in Effectiveness Trials guidelines.
- The study will use a multi-stage process including qualitative interviews, Delphi surveys and a consensus meeting to incorporate multidisciplinary stakeholder perspectives including survivors of critical illness and their family members.
- A limitation of the study is that stakeholders may not be fully representative of all groups.
- There are many possible outcomes for inclusion in the COS, and it may be difficult to reach consensus across stakeholders.

## Introduction

 Following critical illness, patients can experience new or worsening emotional, cognitive and physical problems.^1^,^3^ These problems, which impact up to 60% of survivors, not only affect the individual but can also have a significant impact on the healthcare system and society.^4^ For example, recent evidence has shown that up to 30% of survivors require a hospital readmission within 30 days of discharge, and over 50% of those who were working before admission to critical care do not return to employment at 1 year.^5 6^ As a result of these challenges, family members are often required to adopt the role of informal caregiver.^7^

Family members of survivors of critical illness are known to experience adverse outcomes in the months following the critical care admission.^8 9^ These issues can impact their physical, social and emotional health.^10 11^ For these reasons, research to date has attempted to support family members of survivors of critical illness.^12^ Approaches such as telephone web-based interventions and integrated health and social care programmes have been tested; however, to date, no randomised controlled trial has shown sustained evidence of benefit from any intervention.^13^

One major challenge to the interpretation of existing trial data is the heterogeneity in selection and definition of the outcomes used for evaluation. Often trials which examine similar interventions measure multiple, diverse outcomes. For example, three articles reporting on family outcomes in 2024 used 14 different outcome measures.^14^,^16^ It is evident there is a need for standardisation of outcome selection and definition to ensure future clinical research is effective and efficient.

There has been an increasing international effort to create Core Outcome Sets (COS). These COS derive consensus among people affected by a particular condition and other relevant key stakeholders (patient representatives, clinicians and clinical researchers) as to which outcomes should be reported as a minimum.^17^ A COS is defined as the minimum number of outcomes that should be measured and reported in all clinical trials in a specific field of interest.^18^ The process of developing a COS allows for research that is representative and globally acceptable, and the standardisation of outcome measurement means that more meaningful results can be synthesised to inform clinical practice and care.^19^ Importantly, a COS does not preclude researchers from measuring other outcomes of interest relevant to the specific intervention, including the primary outcome.^20^

At present, no COS for trials has investigated the impact of interventions to improve outcomes for family members of survivors of critical illness. The aim of the Family or Caregiver Outcomes after critical illness (FOCUS) study, therefore, is to develop a COS for trials involving family members or caregivers of survivors of critical illness, where the aim of the intervention is to improve outcomes.

## Methods and analysis

### Registration and approvals

This study is registered on the Core Outcome Measures in Effectiveness Trials (COMET) database (https://comet-initiative.org/Studies/Details/1977).

### Design

**Figure 1:**
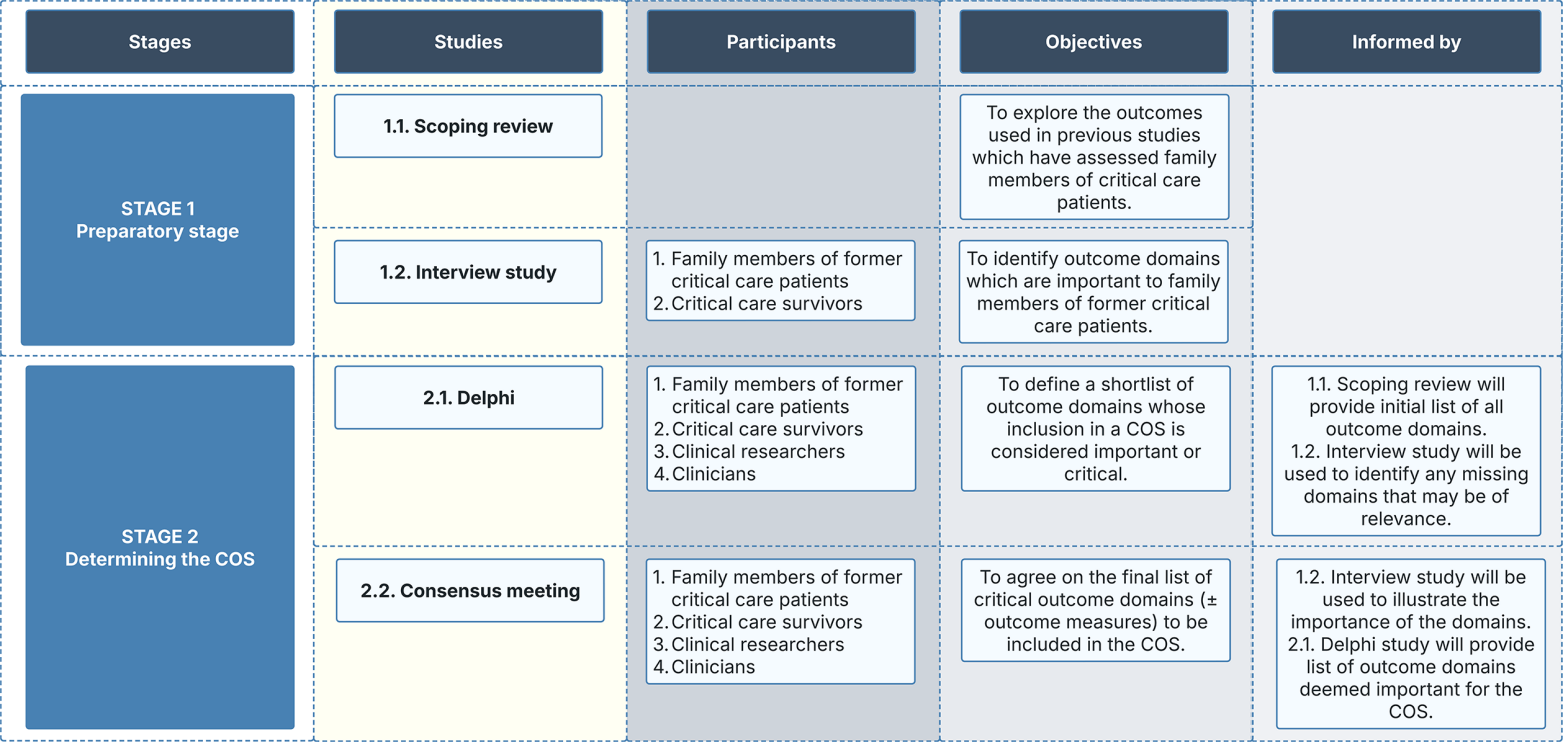
Outline of project components. COS, Core Outcome Sets.

A mixed-methods study adopting an established process for COS development will be utilised.^21^ We will undertake a two-stage process (figure 1). The first preparatory stage (stage 1) will integrate the outcomes of a recently completed scoping review of quantitative literature and qualitative interviews with critical care survivors and family members.^22^ The second stage will involve a consensus process to determine the COS (stage 2). This protocol has been developed in line with guidance from the COMET Handbook, COS Protocol and Development Checklists, and is in keeping with methods adopted by prior critical care-related COS.^23^,^25^

### Study management

A study steering group will be established; this group will consist of study investigators: four clinicians with expertise in the field of critical care, one methodologist with expertise in COS development, two independent experts with relevant research and clinical expertise and two patient/caregiver representatives. The steering group will also include clinicians representing different professional backgrounds (nurses, physicians and physiotherapists) and different specialty areas including neurosurgery, general critical care, care of the older adult and anaesthesia.

The COMET Checklist for Public Research Partners and the COS Study Developers Involved in Designing a COS Study Checklist^25^ will be integrated to facilitate engagement with external steering group members during the study process.

### Patient and public involvement

Patient and public involvement (PPI) has been integral to study design and will continue to guide the study throughout. Our study management group has two patient/caregiver representatives. During study development, regular PPI meetings were held with individuals with lived experience of critical illness (including family members). During the study, PPI will be sought at regular intervals including during the development of lay outcome terms and definitions, providing advice and feedback on survey design, and how and where to disseminate the results to the public through community engagement events and publications.

### Scope of the COS

The scope of the FOCUS study will specifically apply as follows:

1. Research or practice setting: primarily for adoption in clinical research studies evaluating family outcome interventions in the critical care setting, including randomised and controlled trials, comparative study designs with or without controls and observational cohort studies.
2. Health condition: family members or caregivers of patients admitted to critical care. Family members of bereaved patients will be excluded.
3. Population: adult (≥ 18 years of age) family members or caregivers of patients admitted to the critical care. Family members of survivors aged <18 years will be excluded. This COS is intended for adult critical care patients only. To clarify, we have used the term ‘family member’ throughout this protocol; this includes anyone considered to be next of kin or informal caregiver (unpaid) of patients with critical illness.
4. Interventions: any intervention that captures family outcomes according to the domains identified in the recent scoping review.^22^ Timepoints of capturing family outcomes will include either within the intensive care unit (ICU), following ICU discharge or following hospital discharge to the community.

### Stage 1: preparatory stage

This stage will inform the selection of outcomes for round 1 of the modified Delphi process. We will integrate the findings of a recently completed scoping review that explored the measures used in studies examining the outcomes of family members of critical care survivors with semi-structured interviews with families or caregivers.

We have recently completed a qualitative study in relation to outcomes following sepsis and critical illness, which included 53 international interviews with clinicians, survivors and family members.^26^ These interviews explored concepts important during the critical care stay, hospital admission and across the recovery arc, with a specific focus on family members (online supplemental file S1 for interview schedules). To enable meaningful patient and family member input into the development of this COS, we will undertake secondary analysis of the interview transcripts using thematic analysis.^27^ New unique outcomes important to family members will be identified, confirmed as relevant and categorised using the domains identified in the scoping review.^22^

An initial list of outcome measures and outcome domains will be generated from the scoping review and qualitative interviews with patients. Lay descriptions will be provided and developed with the study steering group, including family steering group members. Resources, including plain language summaries provided by the COMET Initiative will be used to introduce the Delphi study. Pilot testing will be undertaken before commencing the formal Delphi survey.

### Stage 2 : determining the COS

Recruitment for the Delphi study will comprise representatives from four key stakeholder groups: clinical researchers, clinicians, critical care survivors and family members and caregivers of critical care survivors. As there is no consensus on the number of participants for a Delphi study, as large a panel as possible for each group will be recruited.^28^ Organisations for each group will be identified using existing networks of national and international critical care contacts and web-based searches. The steering group’s diversity will facilitate recruitment from lower-income, middle-income and high-income countries. Letters of invitation to participate will be emailed to the relevant organisations or directly to an individual outlining the study. This will include timelines for overall completion, estimated time required for each survey round and consent. The COS will be translated into necessary languages to increase generalisability

Only participants responding favourably to the preliminary invitation to participate will be recruited. During the Delphi survey, strategies will be adopted to facilitate retention of participants including personalised invitations; reminders about survey completion; contact details for the lead researcher and regular checks to verify and update contact details. A unique personal number will be assigned to each participant to facilitate monitoring of survey completion. The primary means of contact with participants will be via email, unless other means (eg, telephone) is requested by the participant.

#### Researcher stakeholder group

This group will comprise international experts in critical care research, recruited from previous collaborations and key authors identified in the scoping review.

#### Clinician stakeholder group

Clinicians will be recruited from international professional organisations relevant to critical care, for example, Intensive Care Society (UK) and American Delirium Society. Clinicians will have a primary role in clinical practice and will include all members of the multidisciplinary team.

#### Family and patient stakeholder groups

This group will comprise members of PPI and engagement groups, patient support groups, charities and personal contacts.

#### Modified Delphi methodology for consensus

Participants will score each outcome according to the Grading of Recommendations Assessment, Development, and Evaluation (GRADE) scale.^29^ This ranges from 1 to 9 in terms of importance for inclusion in the final COS:

- 1–3: not important for inclusion
- 4–6: important but not critical
- 7–9: critical for inclusion

Participants will also be provided with an ‘unable to score’ response if they consider themselves unable to rate any outcome and free text boxes to comment. Consensus for inclusion will be defined as ≥70% of responses rating the outcome as a score of ≥7, and ≤15% of responses rating the outcome ≤ 3 on the GRADE scale. Participants will be asked to complete survey rounds within 14 days. Non-respondents will receive weekly reminders, up to 3 weeks after the initial invitation.

##### Round 1

This first questionnaire (round 1) survey will be structured so that the most frequently reported family outcomes as identified in the scoping review^22^ will be listed. This list will be supplemented with outcomes identified in the qualitative interview studies that were either absent or under-represented in frequency in the scoping review despite being highlighted as important by participants in the qualitative interviews. The order of outcomes will be randomised. For each outcome, participants will rate its importance for inclusion in the FOCUS COS. Participants will be able to provide free text comments at the end of the survey. Demographic data (including age, sex, country of residence, duration of clinical and/or research experience and involvement in research) will also be collected to characterise participants.

##### Round 2

Outcomes meeting consensus for ‘not important for inclusion’ will be removed. The remaining outcomes and new outcomes identified from round 1 will be carried forward into round 2. Each participant will be shown their own score from round 1 and will receive feedback on the average scores from each of the stakeholder groups. Participants will be asked to re-score the outcome considering this feedback. Participants will have the opportunity to report reasons for any change of score that alters the overall category of importance rating.

A consensus meeting will be held if the number of outcomes in the final FOCUS COS is considered too high and deemed to be unmanageable for implementation in the clinical research setting. This will be determined by all members of the study steering group. The consensus meeting could potentially help understand when these outcomes should be implemented, an approach utilised in other COS development in the critical care field.^30^

The steering group will first review the findings of the consensus process for establishing the COS to determine the requirements for a consensus meeting and its structure, format and content. Attendees at consensus meetings will comprise clinicians, researchers, family members and patients and will be held virtually to ensure accessibility.

### Strategies for dissemination

Multiple formats of dissemination will be employed including peer-reviewed, open-access publications, presentation at national and international conferences, engagement with journal editors, representatives from national and international funding agencies and policymakers and summaries (lay and professional versions) for circulation through relevant patient and professional (clinical and research) organisations and networks.

### Data capture

This study will be undertaken via REDCap (Research Electronic Data Capture), hosted within the University of Cambridge Integrated Data Environment. REDCap is a web-based application that can be accessed through a web browser on any device with internet access. Participants will be asked to complete basic demographic details and agree to being contacted in a second survey. We will send a letter of thanks and details of the results of the study.

### Study status

Integration of the findings of the scoping review and qualitative interviews will start March 2026. Phase 2 is expected to commence in summer 2026 and the COS ratified by spring 2027. Recruitment to the Delphi process had not started at the time of manuscript submission.

## Discussion

This study protocol presents the methodology for the development of a COS involving family members of patients requiring critical care in line with the COMET Initiative recommendations.^24^

The Delphi technique is extensively used in the literature to reach a consensus.^31^ During a Delphi, a series of questions are sent to a panel of participants with relevant experience, with the aim of reaching a consensus opinion.^23^ The group results are reported back to the panel and often repeated until an agreement has been reached, or an agreement threshold has been achieved.^30^

Developing the COS is the first step in improving the measurement of outcomes in clinical trials in this area. Once the COS is identified, further research will be needed to determine the measurement methods or measurement instruments available for each of the core outcomes, followed by an assessment of the quality and feasibility of using these methods. Development of the core outcome measurement set is beyond the scope of this project. This will allow for recommendations of the most appropriate measurement methods to capture the COS. This process will also require the input of key stakeholders at all stages of the research process.

### Strengths and limitations

This project has several strengths. It is grounded in the rigorous methodological guidelines for core outcome set development provided by the COMET Initiative, and it has been pre-registered in the COMET database.

The project also has limitations. First, although we aim to engage a broad range of stakeholders (eg, through online surveys distributed nationally and internationally by selected international stakeholder groups), participating stakeholders may not be fully representative of all relevant groups because of language barriers and self-selection or volunteer bias. Second, the flexible nature of Delphi studies, including voting across multiple rounds, may lead to participant attrition and introduce bias towards majority opinions.

## Ethics and dissemination

### Ethics

This study has been approved by the University of Cambridge Psychology Research Ethics Committee (Reference: PRE.2025.080). Invitations for participation in this study will be sent through email. This email will provide information about the study including an overview of the purpose of the study and what is involved. Participants will provide written informed electronic consent via a survey link. All participants will have the opportunity to contact the research team to ask questions about the study prior to taking part via an email address provided in the letter of invitation.

### Dissemination

The COS will be shared with multiple stakeholders (eg, clinicians, researchers and research organisations, policymakers and funding agencies), and accessible versions will be developed for public dissemination. The findings will be published in peer-reviewed journals and shared at international conference presentations, as well as through communications via our charity partners (eg, Sepsis Research (UK)). We will work with key stakeholder organisations to promote the COS and its use in relevant clinical research.

## Supplementary material

10.1136/bmjopen-2026-117825 online supplemental file 1
