# Supplementary material for: Family or caregiver outcomes after critical illness (FOCUS): a protocol for a core outcome set development study
Source: BMJ Open. 2026 Jun 23;16(6):e117825. doi: 10.1136/bmjopen-2026-117825 (PMC13295962; doi:10.1136/bmjopen-2026-117825)
Supplement: online supplemental file 1 [file bmjopen-16-6-s001.docx]

### Family member/caregivers

1. Can you tell me a little about the sepsis hospitalisation of your family member/caregiver?
2. Did your family member/caregiver move straight from hospital to home, or did they go to a rehabilitation centre/service/centre? **IF YES,** Can you tell me a little about this experience?
3. Can you tell me about the first few days home after your family member/ caregiver hospital/rehabilitation centre discharge?
4. Did you experience any problems/challenges from a personal perspective following your family members/caregivers sepsis hospitalisation? **IF YES**, can you tell me about challenges from your perspective and how they impacted your day-to-day activities? Did you access any specific support?
5. Did you access/ were you referred to any specialist services for your family members/caregivers recovery from sepsis? Can you tell me about how you accessed these services? Were these services the ‘right fit’ (*explain this term*) for their recovery journey? What could have been improved about this experience? What, if anything was missing?
6. Was your family member/caregiver ever readmitted to the hospital (the same or different hospital) following their initial discharge following sepsis? **IF YES,** Was this readmission related to the sepsis diagnosis? Can you remember the rough timeline of the readmission? Do you think there was anything which could have prevented the readmission? Can you tell me about this? How did it feel to return to the hospital?
7. Did you visit your GP in relation to your family members/caregivers sepsis hospitalisation? **IF YES,** Can you tell me a little more about this visit (or visits)? Was this useful recovery (*prompt: how*)? Did you visit your GP in relation to your own health following the sepsis hospitalisation (*prompt- how was it related*?) Can you tell me a little more about this visit (or visits)? Was this useful in your recovery?
8. Do you think the transition in care (*explain this*) between hospital and community could be improved for sepsis survivors? **IF YES,** how do you think we could improve this experience for sepsis survivors?
9. Do you have concerns related to your family member driving after sepsis? Can you tell us about any concerns and how these might have impacted you and your family?
10. Thinking about the *‘ideal’* sepsis recovery journey for both patients and family members, what from your experience, would this look like?
11. Is there anything about your experience/journey which we have missed?
