# Supplementary material for: Family or caregiver outcomes after critical illness (FOCUS): a protocol for a core outcome set development study
Source: BMJ Open. 2026 Jun 23;16(6):e117825. doi: 10.1136/bmjopen-2026-117825 (PMC13295962; doi:10.1136/bmjopen-2026-117825)
Supplement: Supplementary file 2 [file bmjopen-2026-117825.reviewer_comments.pdf]

PEER REVIEW HISTORY

BMJ Open publishes all reviews undertaken for accepted manuscripts. Reviewers are asked to complete a checklist review form and are provided with free text boxes to elaborate on their assessment. These free text comments are reproduced below.

ARTICLE DETAILS

Title (Provisional)

Family or Caregiver Outcomes after critical illness (FOCUS): a protocol for a core outcome set development study

Authors

Brown, Claire; Hartley, Peter; Malyon, Alexandra; Connolly, Bronwen; Lone, Nazir I; McPeake, Joanne

---

VERSION 1 - REVIEW

**Reviewer**	**1**
**Name**	**Fink, Ericka**
**Affiliation**	**University of Pittsburgh**
**Date**	**01-May-2026**
**COI**	**None**

Brown et al submit methods for a prospective core outcome set for future studies including family or caregiver outcomes. Overall, this topic is important and timely, and is inclusive in stakeholder types, with clear writing. Authors use COMET and COS guidelines, which includes current standards for COS development.

1. p6, line 24. Is consent 'written' or electronic?

2. Study management. The number of study steering group members is reported for some stakeholders (family =2) but not others. Will this group be balanced?

3. p7, line 10. Please clarify if the family steering committee members are also going to provide the feedback throughout the study.

4. Figure 1. More detail on the types of clinicians should be provided (e.g., critical care, PM&R, general surgery, etc).

5. p8, line 52. If the COS is intended to be relevant for international studies, more detail is needed about how you will recruit people from various domains (e.g., clinician, research, families) from low, middle, and high income countries. Please clarify if this is the case and how you intend to ensure it has this amount of generalizability. Will the COS only be in English?

6. p9, line 23. Clarify what professional organizations will be recruited from.

7. p11, line 47. If the COMS is part of this project with the COS, much more information on COMS methods is needed for this manuscript. For example, how will you review instruments and make recommendations.

VERSION 1 - AUTHOR RESPONSE

Reviewer: 1

Comment 1.
p6, line 24. Is consent 'written' or electronic?
Thank you for this comment. We have added:

"Participants will provide written informed electronic consent via a survey link ." (Page 11)

Comment 2.
Study management. The number of study steering group members is reported for some stakeholders (family =2) but not others. Will this group be balanced?

Thank you for noticing this oversight. We have added:

"A study steering group will be established; this group will consist of study investigators; four clinicians with expertise in the field of critical care, one methodologist with expertise in COS development; two independent experts with relevant research and clinical expertise and two patient/caregiver representatives." (Page 5).

Comment 3.
p7, line 10. Please clarify if the family steering committee members are also going to provide the feedback throughout the study.

Thank you for this. We have added:

"Lay descriptions will be provided and developed with the study steering group, including family steering group members." (Page 7).

"This will be determined by all members of the study steering group." (Page 9).

Comment 4.
Figure 1. More detail on the types of clinicians should be provided (e.g., critical care, PM&R, general surgery, etc).

Thank you for this important feedback. We have now included the following:

"Clinicians representing different professional backgrounds (nurses, physicians and physiotherapists) and different speciality areas including neurosurgery, general critical care, care of the older adult and anaesthesia were included in our steering group." (Page 5).

Comment 5.
p8, line 52. If the COS is intended to be relevant for international studies, more detail is needed about how you will recruit people from various domains (e.g., clinician, research, families) from low, middle, and high income countries. Please clarify if this is the case and how you intend to ensure it has this amount of generalizability. Will the COS only be in English?

Thank you for this excellent point. The COS will be translated into the necessary languages, and this process is supported by the software REDCap. We have international stakeholders on the steering group including clinicians from lower middle to income countries who will support dissemination through their networks.

We have added:

"The steering groups diversity will facilitate recruitment from lower, middle and high income countries. Letters of invitation to participate will be emailed to the relevant organisations or directly to an individual outlining the study. This will include timelines for overall completion, estimated time required for each survey round, and consent. The COS will be translated into necessary languages to increase generalisability." (Page 7).

Comment 6.
p9, line 23. Clarify what professional organizations will be recruited from.

Thank you for this comment. We have added:

“Clinicians will be recruited from international professional organisations relevant to critical care, for example Intensive Care Society (UK) and American Delirium Society.” (Page 8).

Comment 7.
p11, line 47. If the COMS is part of this project with the COS, much more information on COMS methods is needed for this manuscript. For example, how will you review instruments and make recommendations.

Thank you for this comment, the COMS is not part of the COS project. We have added:

“Once the COS is identified, further research will be needed to determine the measurement methods or measurement instruments available for each of the core outcomes, followed by an assessment of the quality and feasibility of using these methods. Development of the core outcome measurement set is beyond the scope of this project.” (Page 10)

VERSION 2 - REVIEW

**Reviewer**	**1**
**Name**	**Fink, Ericka**
**Affiliation**	**University of Pittsburgh**
**Date**	**21-May-2026**
**COI**	

Authors have responded adequately to this reviewer's comments.
